# Wild orangutans can simultaneously use two independent vocal sound sources similarly to songbirds and human beatboxers

**DOI:** 10.1093/pnasnexus/pgad182

**Published:** 2023-06-27

**Authors:** Adriano R Lameira, Madeleine E Hardus

**Affiliations:** Department of Psychology, University of Warwick, Coventry CV4 7AL, UK; Independent researcher

**Keywords:** biphonic call combinations, consonant-like calls, vowel-like calls, vocal control, speech evolution

## Abstract

Speech is among the most complex motoric tasks humans ever perform. Songbirds match this achievement during song production through the precise and simultaneous motor control of two sound sources in the syrinx. Integrated and intricate motor control has made songbirds comparative models par excellence for the evolution of speech, however, phylogenetic distance with humans prevents an improved understanding of the precursors that, within the human lineage, drove the emergence of advanced vocal motor control and speech. Here, we report two types of biphonic call combination in wild orangutans that articulatorily resemble human beatboxing and that result from the simultaneous exercise of two vocal sound sources: one unvoiced source achieved through articulatory maneuvering of the lips, tongue, and jaw as typically used for consonant-like call production, plus one voiced source achieved through laryngeal action and voice activation as typically used for vowel-like call production. Orangutan biphonic call combinations showcase unappreciated levels of, and distinct neuromotor channels for, vocal motor control in a wild great ape, providing a direct vocal motor analogy with birdsong based on the precise and simultaneous co-control of two sound sources. Findings suggest that speech and human vocal fluency likely built upon complex call combination, coordination and coarticulation capacities that involved vowel-like and consonant-like calls in an ancestral hominid.

**Significance Statement**

Speech evolution is one of the longest-standing scientific puzzles. Songbirds have been the go-to comparative model for decades to understand the neuro-cognitive transformations involved with and required for advance vocal control. Paradoxically, the vocal capacities of great apes – our closest living relatives – have been underplayed or dismissed, though they are the only realistic proxies of the behaviours that preambled speech within the human lineage. We show that orangutan repertoires include calls that match the motor skill and intricacy of birdsong and human beatboxing. Speech, and probably human song, very likely derived from a set of rich vocal capacities already established among hominid ancestors. Bird-ape collaborative research may help understand what exactly about the hominid body and brain made speech possible.

## Introduction

The neurobiology of speech production involves some of the most complex choreographed movements performed by humans. In nonhuman animals, similar degrees of vocal motoric coordination are notoriously accomplished by songbirds, namely, through the precise motor control of two sound sources in the passerine syrinx during song production (1, 2), each sound source equivalent to one typical set of vocal folds in mammals. Vocal analogy between humans and songbirds (3) cannot, however, elucidate shared ancestry, rendering bird–human functional parallels mute about how or why complex vocal control was implemented by hominid brains in hominid bodies in the process of human ancestors becoming language-able.

Even under the most conservative hypothetical scenario, where spoken language might be considered to have come into existence anew and abruptly in modern humans, one would still expect it to have been directly or indirectly leveraged on the neuromotoric mechanisms already present in prelinguistic hominid ancestors. Using the simile of saltationist hypothesis (4) that language was ignited as if by lightning strike, lightning can only kindle a fire when combustible materials are present where it strikes. Reconstructing the forerunning system of speech among ancestral hominids (5, 6) shall, therefore, remain imperative for explaining why spoken language emerged in the human lineage, irrespective of one's theoretical leanings or alliances.

Here, we report two outstanding vocal behaviors in a wild great ape that represent direct analogies with birdsong production based on two sound sources. This evidence sheds new light on the putative vocal range and complexity of ancient hominids. Furthermore, it may finally allow a reading of birdsong neurobiology from a hominid lens, enabling the exchange of bird–ape comparative knowledge, helping formulate cross-taxa predictions, and ultimately, accelerating the reconstruction of the evolutionary precursors of speech within the Hominid family.

## Results

We observed two types of biphonic call production in wild orangutans (*Pongo* spp), both involving voiceless consonant-like call production (7) “in combination and overlap with” (hereafter “+”) voiced vowel-like call production. These vocal behaviors involved supralaryngeal maneuvering (i.e. of lips, tongue, and/or jaws) together with laryngeal action (i.e. vocal fold oscillation as activated by egressive airflow), respectively. They were, thus, distinct from cases of biphonation as the result of nonlinear oscillations in the mammalian larynx (8) or achieved by merging two formants, as in human throat singing (9).

Simultaneous voiceless + voiced call production involved four known and previously described call types of the orangutan repertoire, which can be produced solo or in combination with other calls (10). They are presumed present across wild populations (10) and do not represent obvious cases of local-specific call types, such as other described orangutan vocal traditions (11).

The first biphonic call combination was composed by “chomps + grumbles” and produced by Bornean flanged male orangutans (*Pongo pygmaeus*) in disturbance and precombat contexts (10) at Tuanan, Central Kalimantan, Indonesian Borneo (see Audio S1). Across 2,510 observation hours, 30 chomps were recorded from two flanged males, and 111 grumbles were recorded from seven flanged males. Among these, there were 16 instances of chomps + grumbles combination by two flanged males (Zeke and Kay).

The second biphonic call combination was composed by “kiss-squeaks + rolling calls” and produced by Sumatran adult female orangutans (*Pongo abelii*) in the context of alarm vocal responses toward predator models at Ketambe, Aceh, Sumatra, Indonesia (12) (see Audio S2). Across 1,287 observation hours, 293 instances of kiss-squeaks + rolling call combinations were recorded from five females (Chris, Elisa, Puji, Sina, and Yet) among a total of 1,176 kiss-squeaks and 1,158 rolling calls from seven adult females.

**Fig. 1.**
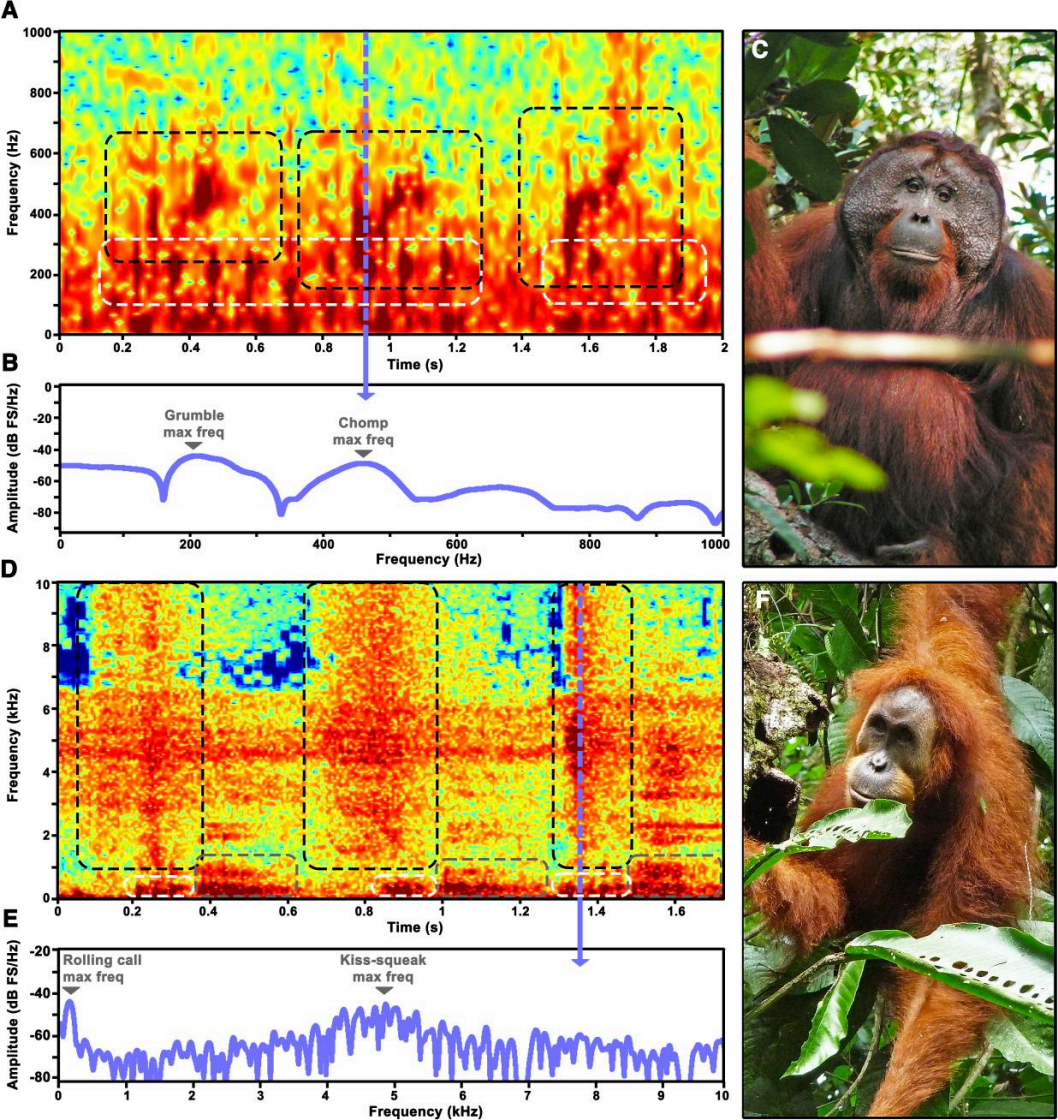
Orangutan biphonic voiceless + voiced call combinations produced by Bornean flanged males (*P. pygmaeus*) and Sumatran adult females (*P. abelii*). A) Spectrographic representation (0- to 40-Hz bandstop filter applied for clarity purposes) of an exemplar case of “chomps + grumbles” by Zeke; dashed boxes highlight general range and profile of chomps (black) and grumbles (white); dashed line (purple) presents time used to extract spectrogram amplitude slice. B) Spectrogram amplitude slice depicting chomp's and grumble's simultaneous frequency maxima. C) Bornean flanged male at Tuanan, Central Kalimantan, Indonesian Borneo (credits: A.R.L.). D) Spectrographic representation of an exemplar case of “kiss-squeaks + rolling calls” by Sina; dashed boxes highlight general range and profile of kiss-squeaks (black), rolling calls (white), and grumphs (gray); dashed line (purple) presents time used to extract spectrogram amplitude slice. E) Spectrogram amplitude slice depicting kiss-squeaks's and rolling call's simultaneous frequency maxima. F) Sumatran adult female at Ketambe, Aceh, Sumatra, Indonesia (credits: M.E.H.).

Figure 1 depicts the spectrographic representation of exemplar cases of the two identified biphonic call combinations (Fig. 1A and D), with inspection by ear providing a clear sense of the double nature of these combinations’ sound source (see Audios S1 and S2). Given the different nature of the two sound sources (one in the mouth and one in the larynx), the result was two concurrent, yet distinct sound profiles for each biphonic call combination. Voiceless calls exhibited a higher register in the frequency spectrum and voiced calls a lower register. For “chomps + grumbles” combinations, chomps represent “bubbly” calls and exhibit elements that ascend along a frequency slope between ∼300–600 Hz for ∼0.20 s (10), whereas grumbles resemble a starting engine and exhibit strings ∼1.0 s long of staccato elements at ∼185 Hz (10), each element with ∼0.045 s. Note the independent production and coordination of three chomps along two grumbles (Fig. 1A).

Kiss-squeaks + rolling calls exhibited a more contrasting difference between the two sources, where kiss-squeaks (articulatorily and acoustically homologous to a human kiss-sound) exhibit a stark onset with a noisy follow through composed by a wideband of acoustic energy spreading ∼3,850 Hz for ∼0.375 s (10), whereas rolling calls exhibit elements with acoustic energy concentrated around ∼240 Hz, stringed into sequences ∼0,325 s long (10), where each element is ∼0.03 s long. Note that these biphonic combinations were part of larger call sequences that included other voiced call types, e.g. “grumphs” (10) (Fig. 1D). Spectrogram slice view (Fig. 1D and E) showed two simultaneous frequency maxima for each biphonic call combination, confirming simultaneous production.

## Discussion

Findings reveal that complex motor control involving the combination and coarticulation of two sound sources is found in one of our closest living relatives, wild orangutans. We report and describe two cases of biphonic call production in adult males and females across contexts and across different species of *Pongo*. This confirms that these vocal behaviors have a common biological basis and were not a simple spurious observation.

Due to their intrinsic nature, biphonic call combinations in orangutans imply distinct channels for neuromotor vocal control, demonstrating new and hitherto underestimated vocal control and coordination capacities in a wild great ape. Based on shared ancestry within the Hominid family, this discovery shows that similar homologous vocal behaviors may have been present in an ancient, now-extinct nonhuman hominid ancestor and that similar or equivalent capacities likely propped speech evolution.

Similar homologous biphonic behavior in humans, involving synchronous supralaryngeal and laryngeal sound production, is found in beatboxing (13). Simultaneously, orangutan biphonic call combinations are analogous to birdsong, also governed by two sound sources (1). These parallels with human and bird vocal expression align with cumulating evidence for advanced vocal control in great apes that challenge traditional assumptions (5, 14).

Orangutan biphonic production also seems in part *functionally* analogous to birdsong and homologous to human beatboxing, where vocal complexity and “exuberance” appear to signal fitness, vigor, and/or condition. For example, chomps + grumbles were produced by male orangutans toward challengers, much like birdsong can be exchanged between male rivals and how beatboxing is used in “battles” between vocal performers. This function would also parsimoniously explain kiss-squeaks + rolling calls by females toward predators, with more complex vocal displays predicted to dissuade more effectively a predator attack.

A behavior's function is not per se an indication of its means of acquisition. For example, some birdsong motifs are innate (15), but this doesn’t take away the fact that song production is nonetheless motorically complex in these cases. As such, further study will be required to elucidate the development of biphonic production in orangutans. However, homology with human beatboxing and cases of vocal learning in great apes (5, 14) hint at a potential role of practice and auditory feedback.

Birdsong represents a case of convergent vocal evolution with humans, however, analogy across two lineages does not inform homology within one. Accordingly, it has been so far unclear how the abundant knowledge gathered for decades on songbird models (3) can be “translated” into the brains and bodies of ancestral hominids for a true-to-life reconstruction of speech evolution. Our findings show that, beyond humans, birdsong is also convergent with some vocal behaviors in great apes. If songbird’s neurobiology for vocal control is analogous to humans’ (3), it follows that it must also be analogous to great apes’, at least in some degree. This indicates that bird-to-ape exchange of knowledge is possible and desirable. For example, in-depth understanding of birdsong neural and molecular substrates could help develop and test predictions about parallel mechanisms in the great ape brain and bodies, avoiding invasive and ethically prohibitive research in great apes. New bird-inferred insight into great ape neurobiology could also guide fieldworkers and primatologists focus empirical and logistical effort on certain contexts or individual classes. This could potentially help advance more expeditiously the cataloguing of great ape vocal behavior that is currently eroding in the wild (16) and in populations that face increasing extinction risks (17). This concerted effort could assist the identification of further yet-undetermined bird–ape vocal analogies.

Our findings align vocal research in birds and hominids (both human and nonhuman) in ways thus far unseen. They invite comparative and cognitive sciences to collaborate and “share brains” in new ways, heralding leaps in bird–ape cross-pollinating studies and the understanding of speech (and potentially song) evolution in the human clade.

## Materials and methods

### Data collection

Data collection involved opportunistic and experimental (i.e. during predator model experiments) audio recordings of orangutan vocal behavior over 2,510 observations hours at Tuanan (2°09′S; 114°26′E) (10) and 1,287 observation hours at Ketambe (3°41′N, 97°39′E) (12). Data collection involved no interaction with or handling of animals. Research was approved by Indonesian authorities and strictly followed the Indonesian law and local guidelines.

### Data analyses

Spectrogram representation and spectrogram slices of orangutan biphonic call combinations were built using Raven Pro 1.6 (18) using window type: Hann; 3-dB filter bandwidth: 56.3 Hz; grid frequency resolution: 1.46 Hz; and grid time resolution: 1,126 samples, according to published protocols (19). No sound or file transformation was applied to avoid interpretation or verification issues. To ascertain biphonic vocal production, we identified, audibly and through spectrogram inspection, instances when two sound sources were patently present.

Biphonic call combination was subsequently confirmed by identifying the power for each independent sound source in the spectrogram slice view during moments of simultaneous sound production. This was possible because consonant-like and vowel-like calls are inherently underpinned by distinct production mechanisms and thus produce distinct acoustic profiles. The original sound files can be found as supplementary materials to facilitate open and transparent inspection of biphonic call production. Given that laryngeal voice production is not patently detectable through video (e.g. as opposed to lip action to produce consonant-like calls), sound analyses provided the most reliable means to establish biphonism. Other setups for the detection of sound location in living mammals require animals of substantial size (e.g. elephants) in captivity (20). Similar setups (e.g. involving an array of 48 microphones arranged in a specific shape around a central video camera) would be practically impossible in a rainforest, with an arboreal species, with an animal with the body size of an ape and/or at a distance.

For information about acoustic variation in the call types comprising biphonic combinations, including individual, age–sex, contextual, and geographic acoustic variations, please see Hardus et al. and Lameira et al. (10, 19). Please note that these levels of variation carry no theoretical or mechanical consequence on the demonstration of biphonism (e.g. a call type may be produced biphonically whether it exhibits geographic variation or not).

## Supplementary Material

pgad182_Supplementary_Data

## Data Availability

All data needed to evaluate the conclusions in the paper are present in the paper and Supplementary materials.

## References

[1] Garcia SM , et al 2017. Evolution of vocal diversity through morphological adaptation without vocal learning or complex neural control. Curr Biol. 27:2677–2683.e3. 28867206 10.1016/j.cub.2017.07.059 PMC5599221

[2] Elemans CPH , et al 2015. Universal mechanisms of sound production and control in birds and mammals. Nat Commun. 6:8978. 26612008 10.1038/ncomms9978 PMC4674827

[3] Jarvis ED . 2019. Evolution of vocal learning and spoken language. Science 366:50–54. 31604300 10.1126/science.aax0287

[4] Chomsky N , Mukherji N. 2009. The architecture of language, 5.impression. Oxford, UK: Oxford University Press.

[5] Lameira AR . 2017. Bidding evidence for primate vocal learning and the cultural substrates for speech evolution. Neurosci Biobehav Rev. 83:429–439. 28947156 10.1016/j.neubiorev.2017.09.021

[6] Lameira AR , Call J. 2020. Understanding language evolution: beyond *Pan*-centrism. BioEssays 42:1900102. 10.1002/bies.201900102 31994246

[7] Lameira AR , Maddieson I, Zuberbuhler K. 2014. Primate feedstock for the evolution of consonants. Trends Cogn Sci. 18:60–62. 24238780 10.1016/j.tics.2013.10.013

[8] Townsend SW , Manser MB. 2011. The function of nonlinear phenomena in meerkat alarm calls. Biol Lett. 7:47–49. 20659926 10.1098/rsbl.2010.0537 PMC3030881

[9] Bergevin C , et al 2020. Overtone focusing in biphonic Tuvan throat singing. eLife 9:e50476. 10.7554/eLife.50476 PMC7064340 32048990

[10] Hardus ME , et al A description of the orangutan's vocal and sound repertoire, with a focus on geographic variation. 2009. In: Wich S. Setia MT. Utami SS, Schaik C, editors. Orangutans. Oxford, UK: Oxford University Press. p. 49–60.

[11] Wich SA , et al 2012. Call cultures in orang-utans? PLoS One 7:e36180. 10.1371/journal.pone.0036180 PMC3346723 22586464

[12] Lameira AR , Call J. 2018. Time-space–displaced responses in the orangutan vocal system. Sci Adv. 4:eaau3401. 10.1126/sciadv.aau3401 PMC6235548 30443595

[13] Dehais-Underdown A , Vignes P, Crevier-Buchman L, Demolin D. 2021. In and out: production mechanisms in human beatboxing:060005. Proceedings of Meetings on Acoustics. 45.

[14] Lameira AR , Hardus ME, Mielke A, Wich SA, Shumaker RW. 2016. Vocal fold control beyond the species-specific repertoire in an orang-utan. Sci Rep. 6:30315. 10.1038/srep30315 PMC4962094 27461756

[15] Fehér O , Wang H, Saar S, Mitra PP, Tchernichovski O. 2009. De novo establishment of wild-type song culture in the zebra finch. Nature 459:564–568. 19412161 10.1038/nature07994 PMC2693086

[16] Kühl HS , et al 2019. Human impact erodes chimpanzee behavioral diversity. Science 363:1453–1455. 30846610 10.1126/science.aau4532

[17] Estrada A , et al 2017. Impending extinction crisis of the world's primates: why primates matter. Sci Adv. 3:e1600946. 10.1126/sciadv.1600946 PMC5242557 28116351

[18] K. Lisa Yang Center for Conservation Bioacoustics, Raven Pro: Interactive Sound Analysis Software. 2023.

[19] Lameira AR , et al 2017. Proto-consonants were information-dense via identical bioacoustic tags to proto-vowels. Nat Hum Behav. 1:0044.

[20] Beeck VC , Heilmann G, Kerscher M, Stoeger AS. 2022. Sound visualization demonstrates velopharyngeal coupling and complex spectral variability in Asian elephants. Animals (Basel) 12:2119. 36009709 10.3390/ani12162119 PMC9404934

